# Association between habitual MSG consumption and compulsive eating behaviors in university students: A cross-sectional study

**DOI:** 10.1371/journal.pone.0360433

**Published:** 2026-11-04

**Authors:** Lina K. Al Jabry, Abdallah Shawwa, Yomna Sarhan, Rola Issa, Omar Abweh, Haneen Qashqeesh, Hazem Alagha

**Affiliations:** 1 College of Medicine, Hebron University, Hebron, Palestine; 2 School of Medicine, University of Jordan, Amman, Jordan; 3 Faculty of Medicine, An-Najah National University, Nablus, Palestine; Guangdong Nephrotic Drug Engineering Technology Research Center, Institute of Consun Co. for Chinese Medicine in Kidney Diseases, CHINA

## Abstract

**Background:**

Monosodium glutamate (MSG), a widely used flavor enhancer, increases food palatability and may influence eating behavior. Although considered safe from a toxicological perspective, its potential association with compulsive eating patterns remains unclear. University students are particularly vulnerable to irregular dietary habits and high consumption of ultra-processed foods.

**Methods:**

A cross-sectional study was conducted among 555 Palestinian university students aged 18–30 years. MSG intake was assessed using a composite dietary exposure scale and categorized into tertiles (low, moderate, high). Food addiction was measured using the Yale Food Addiction Scale (YFAS), binge-eating severity using the Binge Eating Scale (BES), and perceived stress using the Perceived Stress Scale–4 (PSS-4). Logistic regression examined associations between MSG intake and food addiction, while linear regression assessed relationships with binge-eating severity and YFAS symptom counts, adjusting for age, gender, BMI, perceived stress, and academic year.

**Results:**

Food addiction was present in 24.7% of participants and increased across MSG tertiles (15.7% low vs. 33.0% high; p < 0.001). High MSG intake was associated with increased odds food addiction (adjusted OR = 2.75, 95% CI 1.63–4.65, p < 0.001). Higher MSG consumption was associated with greater binge-eating severity (β = 1.25, p < 0.001) and increased YFAS symptom counts, indicating a dose–response relationship. BMI and perceived stress were independent predictors of both outcomes. The adjusted model showed acceptable discrimination (area under the receiver operating characteristic curve [AUC] = 0.71).

**Conclusions:**

Higher MSG intake was associated with increased food addiction symptoms and binge-eating severity among university students, suggesting frequent consumption of flavor-enhanced foods is a correlate of reward-driven eating behaviors, warranting further investigation.

## Introduction

Monosodium glutamate (MSG) appears as a white, salt-like powder. Glutamate, its active ingredient, is a non-essential amino acid naturally found in many commonly consumed foods, such as meat and seaweed. MSG, the sodium salt of glutamate, became commercially available after the isolation, characterization, and subsequent synthesis of the umami compound in the early 20th century [1]. It is now widely used as a food additive to reduce cooking time and enhance the sensory properties of ready-made and processed foods. Furthermore, excessive consumption of MSG has been associated with various health problems, including oxidative stress, neurotoxicity, and metabolic disorders in animal models [2].

MSG is one of the most common flavor enhancers, particularly in processed Asian and Western foods. Its flavor-enhancing properties have made it a widely used additive in soups, snacks, and ready meals, and it is widely consumed by various segments of society, including university students [3]. Despite its prevalence, general knowledge regarding the effects of MSG and its potential long-term impact on eating habits remains limited, necessitating further investigation into its role in modern dietary patterns [4].

Compulsive eating behavior, known as binge eating disorder (BED), is characterized by recurrent episodes of consuming large amounts of food in a limited period of time, accompanied by a sense of loss of control. According to many studies, university students tend to have a high prevalence of eating disorders compared with the general population, which can influence the students’ physical and mental health as well as their academic performance [5–9].

Neurobiologically, dysregulation of dopaminergic reward pathways is involved in binge eating disorders, which may lead to compulsive consumption of palatable foods. Research indicates that large consumption of highly palatable and processed foods is associated with reward pathways, which may increase the frequency of binge eating disorders [10]. Since MSG enhances palatability and is used in processed food, it may contribute to the activation of reward pathways and thereby reinforce compulsive eating behavior [4].

However, research confirming the relationship between MSG and compulsive eating behavior remains limited. Therefore, this study aimed to examine the relationship between MSG consumption and eating behavior among university students. To our knowledge, this is among the first studies to investigate MSG exposure as a behavioral dietary factor associated with addictive-like eating patterns in a Middle Eastern university population.

## Methods

### Study design and setting

In this study, we conducted a cross-sectional analytical study to evaluate the association between habitual MSG consumption and compulsive eating behaviors amongst university students in Palestine. Data was collected using an online, structured, self-administered questionnaire developed on Google Forms. A link to the questionnaire was initially shared with university students via social media platforms (WhatsApp, Instagram, and university student group pages). Participants were then encouraged to forward the link to fellow students in their networks, constituting a non-probability snowball sampling approach to maximize reach across different universities and academic years.

Data was collected between 25 November 2025 and 23 December 2025, as indicated by Google Forms timestamps. A total of 555 university students fully completed the questionnaire and were included in the final analysis.

### Participants

In this study, we included current university students living in the West Bank, Palestine. Eligible participants were required to meet all of the following inclusion criteria: to be currently enrolled as a university student, to report regular MSG intake, which was assessed and later categorized into tertiles (low, moderate, and high) based on the composite exposure score.

Participants were excluded if they met any of the following criteria: not currently enrolled as a university student at the time of data collection, or a self-reported previous or current diagnosis of an eating disorder, or refusal or inability to provide informed consent to participate in the study.

To maintain the integrity of the dataset, statistical analyses were restricted to questionnaires that had been completed in full. A total of 555 filled questionnaires were analyzed. Participants in this study had a mean age of 21.1 ± 2.0 years, with ages ranging from 18 to 30 years. The majority of respondents were female (60.2%), with males comprising (39.8%). In the final sample, university students were distributed across all academic years, with the highest proportion in the fourth year (23.2%), followed by the third year (20.9%). Approximately one-quarter of the sample met criteria for food addiction (24.7%). Moderate and severe binge eating were reported by 25.2% and 4.1% of participants, respectively.

### Data collection tool and procedure

A structured, self-reported questionnaire was developed in both Arabic and English to meet the research objectives. The questionnaire was initially drafted in English and then translated into Arabic using a forward-backward translation procedure: a bilingual researcher translated the English version into Arabic, and an independent bilingual translator back-translated it into English. Discrepancies between the original and back-translated versions were reviewed and resolved by the research team to ensure semantic equivalence.

The questionnaire consisted of five sections as follows: the first section included demographic and anthropometric characteristics: Age, gender, college, university year of study, self-reported height, and weight (for BMI calculation), the second section assessed the frequency of MSG consumption using a Food Frequency Questionnaire (FFQ) focusing on 20 common MSG-rich processed foods, the third section evaluated symptoms of food addiction based on DSM-5 criteria using the Yale Food Addiction Scale 2.0 (YFAS 2.0) [11], the fourth section assessed the presence and severity of binge eating behaviors using the Binge Eating Scale (BES) [12], and the fifth section measured perceived stress levels over the past month using the Perceived Stress Scale (PSS-4) [13]. The questionnaire can be found in S1 File.

In order to ensure the validity of the questionnaire, items were adapted from previously validated instruments commonly used in nutritional and behavioral research. The questionnaire was reviewed by experts in public health, nutrition, and psychology in order to assess clarity, relevance, and comprehensiveness. A pilot study was conducted among 20 university students aged 18–25 to evaluate feasibility and reliability, and minor modifications were made based on participant feedback. The pilot confirmed item comprehension and questionnaire flow; minor wording modifications were made based on participant feedback.

The questionnaire was distributed online to university students, and data was entered and managed using Google Spreadsheets. Variables were predefined and documented by the research team, and data quality was ensured through verification procedures, including double-checking for inconsistencies and missing values. Data collection began in 11/25/2025 and ended in 12/23/2025.

### Statistical analysis

All statistical analyses were performed using R software (version 4.5.1). A complete data audit was conducted before inferential testing, including detection of duplicate entries, verification of variable coding, assessment of missingness, and range checks for anthropometric and psychometric variables. Continuous variables were summarized as means with standard deviations, while categorical variables were reported as frequencies and percentages.

Participants were categorized into tertiles of the composite MSG intake score (low, moderate, and high) based on equal distribution of the sample. Baseline characteristics across tertiles were compared using one-way analysis of variance (ANOVA) for continuous variables and chi-square tests for categorical variables.

The primary outcome was food addiction, defined according to the Yale Food Addiction Scale (YFAS) criteria. The association between MSG intake and food addiction was assessed using logistic regression models. Odds ratios (ORs) with 95% confidence intervals (CIs) were calculated. A fully adjusted model was constructed, including age, gender, body mass index (BMI), perceived stress score (PSS-4), and academic year as covariates. In addition to tertile-based analyses, MSG intake was also modeled as a continuous variable to evaluate dose–response relationships. Sensitivity analyses were performed using alternative model specifications to assess the robustness of the findings.

Secondary outcomes included binge-eating severity, measured by the Binge Eating Scale (BES), and food addiction symptom burden, measured as the YFAS symptom count. These outcomes were analyzed using multivariable linear regression models with the same adjustment set as the primary analysis. Regression coefficients (β) with standard errors (SEs) and p-values were reported. Model fit was assessed using adjusted R^2^ values.

Model assumptions were evaluated using standard diagnostic procedures. For linear regression models, residual plots, Q–Q plots, scale–location plots, and leverage plots were inspected to assess normality, homoscedasticity, and the presence of influential observations. For the logistic regression model, discrimination was evaluated using the area under the receiver operating characteristic curve (AUC), and calibration was assessed using calibration plots and the Hosmer–Lemeshow goodness-of-fit test.

Internal consistency of the MSG composite, BES, and PSS-4 scales was evaluated using Cronbach’s alpha coefficients. Construct validity was assessed using Spearman correlation coefficients between MSG intake, BES scores, YFAS symptom count, and perceived stress.

All statistical tests were two-sided, and a p-value < 0.05 was considered statistically significant.

### Ethical considerations

This study was conducted in accordance with the Declaration of Helsinki and applicable local regulations. Participation in this study was entirely voluntary and anonymous, as subject confidentiality was strictly maintained, and informed consent was obtained at the start of the questionnaire. Participants were informed that the information they gave would be strictly confidential. All data were securely stored, and access was only given to members of the research team, and results were reported in an aggregated form. Ethical approval was granted by the Institutional Review Board of Hebron University College of Medicine. Reference number: ER.CM.68.2025.The anonymized dataset supporting the findings of this study is available as Supporting Information in S2 File. All personally identifiable information has been removed in accordance with participant consent and applicable data protection guidelines.

## Results

### Participant characteristics

A total of 560 survey responses were initially received. After identification and removal of five exact duplicate records, the final analytic sample consisted of 555 participants. Missingness was minimal for key variables, with only two missing age values (0.36%) and one missing height value (0.18%), while all other demographic, anthropometric, and psychometric variables were complete.

Participants were young adults aged 18–30 years, with a mean age of 21.1 ± 2.0 years and a mean BMI of 23.6 ± 4.1 kg/m^2^. Females comprised 60.2% of the sample, while males represented 39.8%. Based on YFAS criteria, 24.7% of participants met the operational definition of food addiction. According to the Binge Eating Scale, 70.6% had no binge-eating symptoms, 25.2% had moderate symptoms, and 4.1% had severe symptoms. The mean perceived stress score (PSS-4) was 7.24 ± 3.19.

These baseline characteristics are summarized in Table 1.

**Table 1:** Baseline demographic, anthropometric, and eating behavior characteristics of university students.

Variable	Overall
**Age, years**	21.1 ± 2.0
**BMI, kg/m²**	23.6 ± 4.1
**PSS-4 score**	7.24 ± 3.19
**MSG composite score**	19.8 ± 8.5
**Gender**	
Female	334 (60.2%)
Male	221 (39.8%)
**Study year**	
Year 1	69 (12.4%)
Year 2	74 (13.3%)
Year 3	116 (20.9%)
Year 4	129 (23.2%)
Year 5	100 (18.0%)
Year 6	67 (12.1%)
**Food addiction (YFAS)**	
No	418 (75.3%)
Yes	137 (24.7%)
**Binge eating (BES)**	
No binge eating	392 (70.6%)
Moderate	140 (25.2%)
Severe	23 (4.1%)

### Baseline characteristics by MSG intake tertile

Participants were categorized into equal tertiles of MSG exposure (low, moderate, and high; n = 185 each). Age, BMI, perceived stress, gender distribution, and academic year were comparable across tertiles, with no statistically significant differences observed (all p > 0.05).

In contrast, significant differences were observed in eating-related outcomes. The prevalence of YFAS-defined food addiction increased stepwise across tertiles, rising from 15.7% in the low tertile to 25.4% in the moderate tertile and 33.0% in the high tertile (p = 0.00056). Similarly, binge-eating severity differed significantly across tertiles (p = 0.00069), with the proportion of participants without binge-eating symptoms decreasing from 78.4% in the low tertile to 59.5% in the high tertile.

These comparisons are presented in Table 2.

**Table 2:** Comparison of demographic, anthropometric, and eating behavior characteristics across MSG intake tertiles.

Variable	Low	Moderate	High	p-value
**N**	185	185	185	—
Age (years)	21.09 ± 2.01	20.99 ± 1.91	21.15 ± 2.07	0.834
BMI (kg/m²)	23.55 ± 4.28	23.92 ± 4.11	23.36 ± 4.03	0.343
PSS-4 score	6.93 ± 3.29	7.25 ± 3.30	7.53 ± 2.95	0.131
MSG total score	11.49 ± 3.18	18.55 ± 1.91	29.29 ± 6.68	—
**Gender**				0.618
Female	114 (61.6%)	106 (57.3%)	114 (61.6%)	
Male	71 (38.4%)	79 (42.7%)	71 (38.4%)	
**Food addiction (YFAS)**				**0.00056**
No	156 (84.3%)	138 (74.6%)	124 (67.0%)	
Yes	29 (15.7%)	47 (25.4%)	61 (33.0%)	
**BES category**				**0.00069**
No binge eating	145 (78.4%)	137 (74.1%)	110 (59.5%)	
Moderate	33 (17.8%)	44 (23.8%)	63 (34.1%)	
Severe	7 (3.8%)	4 (2.2%)	12 (6.5%)	

### Association between MSG intake and food addiction diagnosis

In unadjusted logistic regression analyses, participants in the moderate MSG tertile had significantly higher odds of food addiction compared with those in the low tertile (OR = 1.89, 95% CI 1.12–3.17, p = 0.017). The association was stronger in the high tertile, where participants exhibited nearly threefold higher odds of food addiction (OR = 2.75, 95% CI 1.65–4.55, p < 0.001), indicating a substantial increase in risk compared with the low MSG intake group.

After adjustment for age, gender, BMI, perceived stress, and study year, the association remained statistically significant. Compared with the low tertile, the moderate tertile was associated with increased odds of food addiction (aOR = 1.79, 95% CI 1.04–3.07, p = 0.034), while the high tertile showed nearly threefold higher odds (aOR = 2.75, 95% CI 1.63–4.65, p < 0.001).

When MSG exposure was modeled as a continuous variable, each one-unit increase in the composite score was associated with a 4% increase in the odds of food addiction (aOR = 1.04, 95% CI 1.02–1.06, p = 0.001).

These regression findings are summarized in Table 3 and visually illustrated in Fig 1, which presents the adjusted odds ratios from the fully adjusted model.

**Table 3:** Association between MSG intake and food addiction.

Variable	OR	95% CI	p-value
**Unadjusted**			
Moderate vs Low	1.89	1.12–3.17	0.017
High vs Low	2.75	1.65–4.55	<0.001
Adjusted*			
Moderate vs Low	1.79	1.04–3.07	0.034
High vs Low	2.75	1.63–4.65	<0.001

**Fig 1:**
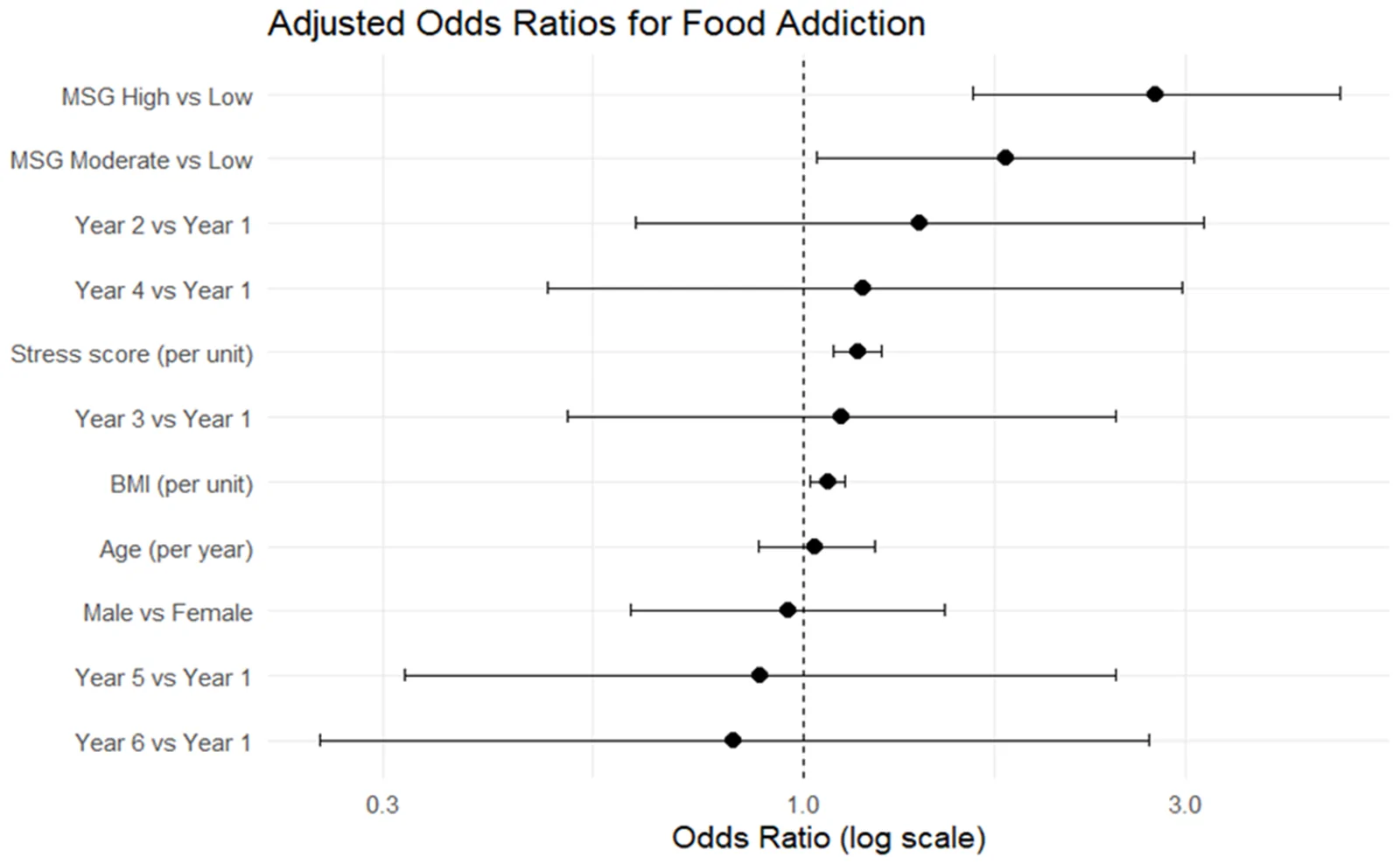
Adjusted odds ratios for food addiction according to MSG intake tertiles. Forest plot displaying odds ratios and 95% confidence intervals from the multivariable logistic regression model adjusted for age, gender, BMI, perceived stress score, and study year. Among covariates, both BMI and perceived stress were independently associated with greater odds of food addiction, while age, gender, and study year were not significant predictors.

### Sensitivity analyses

Sensitivity analyses using multiple model specifications yielded consistent results. Across unadjusted, minimally adjusted, BMI-excluded, and fully adjusted models, the moderate MSG tertile was associated with approximately 1.8–1.9-fold higher odds of food addiction, while the high tertile was associated with approximately 2.6–2.8-fold higher odds compared with the low tertile.

The stability of these effect estimates across models suggests that the observed association between MSG intake and food addiction is robust and not driven by any single covariate.

### Association between MSG intake and binge-eating severity

In multivariable linear regression analysis using the BES total score as the outcome, higher MSG intake was associated with greater binge-eating severity. Compared with the low tertile, participants in the high MSG tertile had significantly higher BES scores (β = 1.25, SE = 0.24, p < 0.001). In contrast, the moderate tertile did not differ significantly from the reference group (β = 0.09, SE = 0.24, p = 0.70).

BMI and perceived stress were also significant predictors of binge-eating severity. The model explained approximately 21% of the variance in BES scores (adjusted R² = 0.214).

Model diagnostics are shown in Fig 2, which demonstrate acceptable adherence to linear regression assumptions, with no major outliers or influential observations.

**Fig 2:**
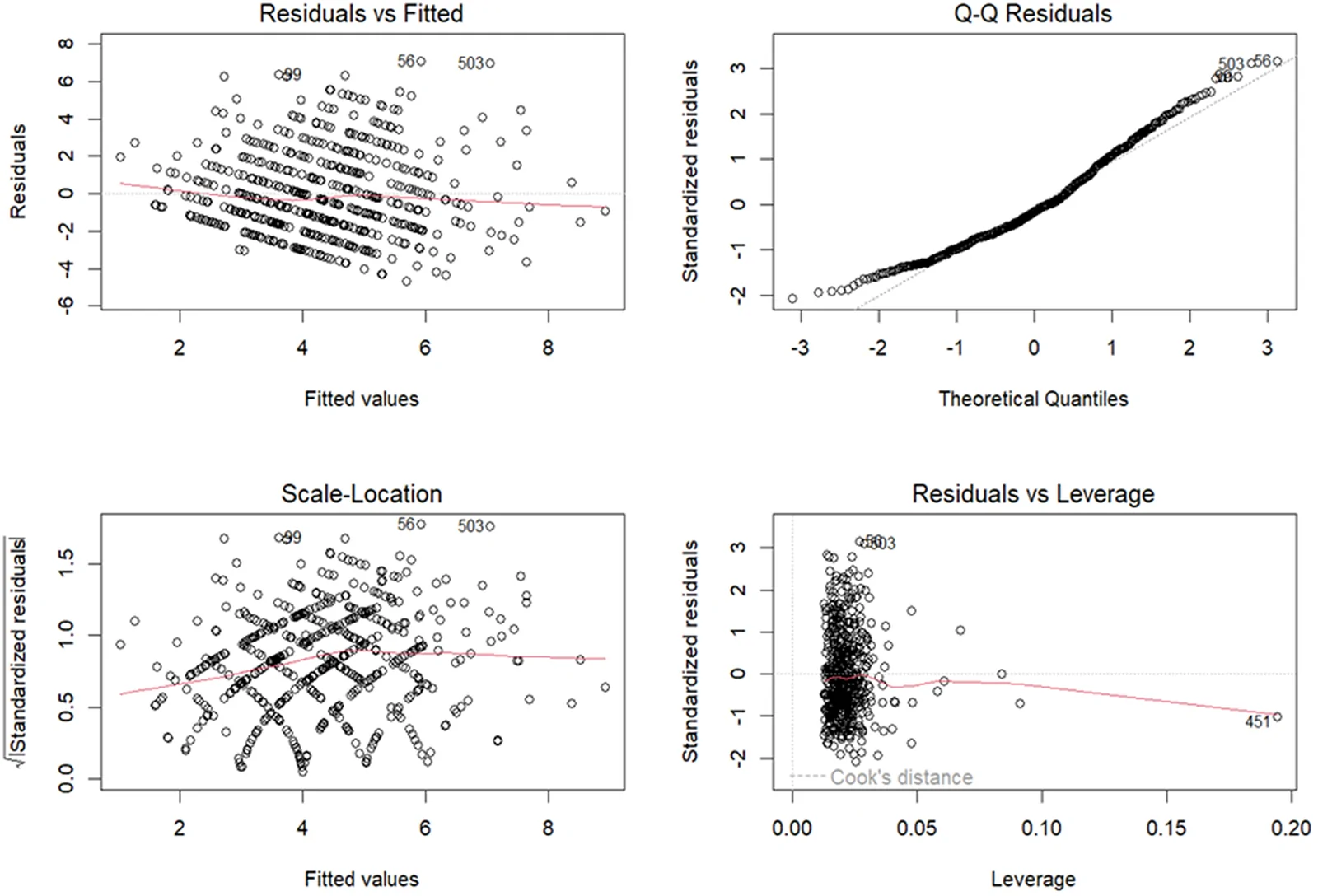
Diagnostic plots for the linear regression model predicting binge-eating severity (BES total score). Panels show residuals versus fitted values, normal Q–Q plot, scale–location plot, and residuals versus leverage, indicating acceptable model fit.

### Association between MSG intake and food addiction symptom burden

In linear regression analysis using YFAS symptom count as a continuous outcome, higher MSG intake demonstrated a clear dose–response relationship. Compared with the low tertile, participants in the moderate tertile exhibited significantly higher symptom counts (β = 0.97, p = 0.001), while those in the high tertile showed a substantially greater increase (β = 2.79, p < 0.001).

BMI and perceived stress were also independently associated with higher symptom counts. The model explained approximately 22% of the variance in symptom burden (adjusted R² = 0.218).

Diagnostic plots for this model are presented in Fig 3, showing generally acceptable model assumptions with only mild heteroscedasticity.

**Fig 3:**
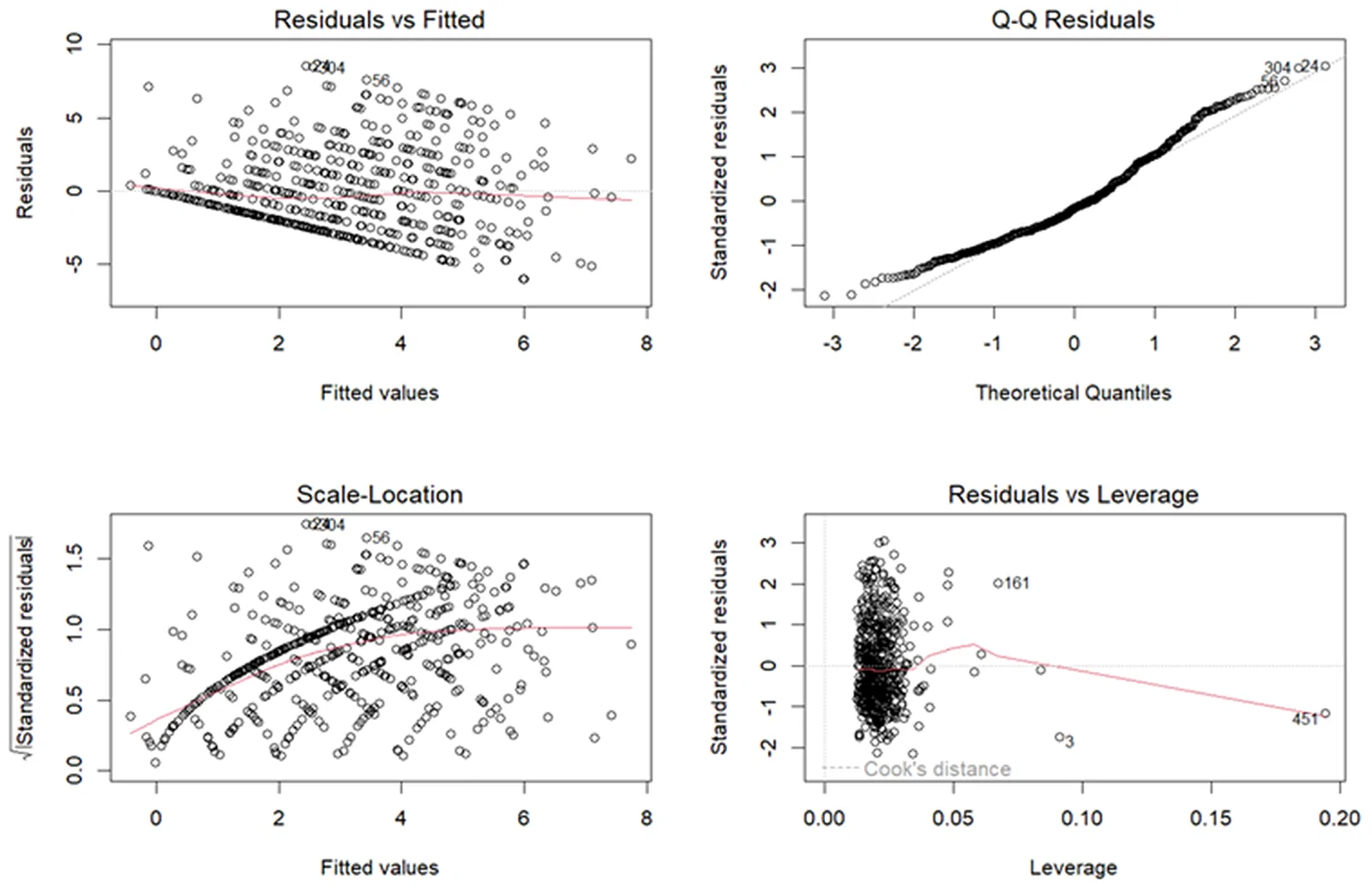
Diagnostic plots for the linear regression model predicting YFAS symptom count. Panels show residuals versus fitted values, normal Q–Q plot, scale–location plot, and residuals versus leverage, indicating acceptable model assumptions.

### Model performance and validation

The adjusted logistic regression model demonstrated acceptable discriminatory performance, with an area under the receiver operating characteristic curve (AUC) of 0.71. Calibration analysis showed good agreement between predicted and observed probabilities, and the Hosmer–Lemeshow test indicated adequate model fit (p = 0.36). The ROC curve for the adjusted model is shown in Fig 4.

**Fig 4:**
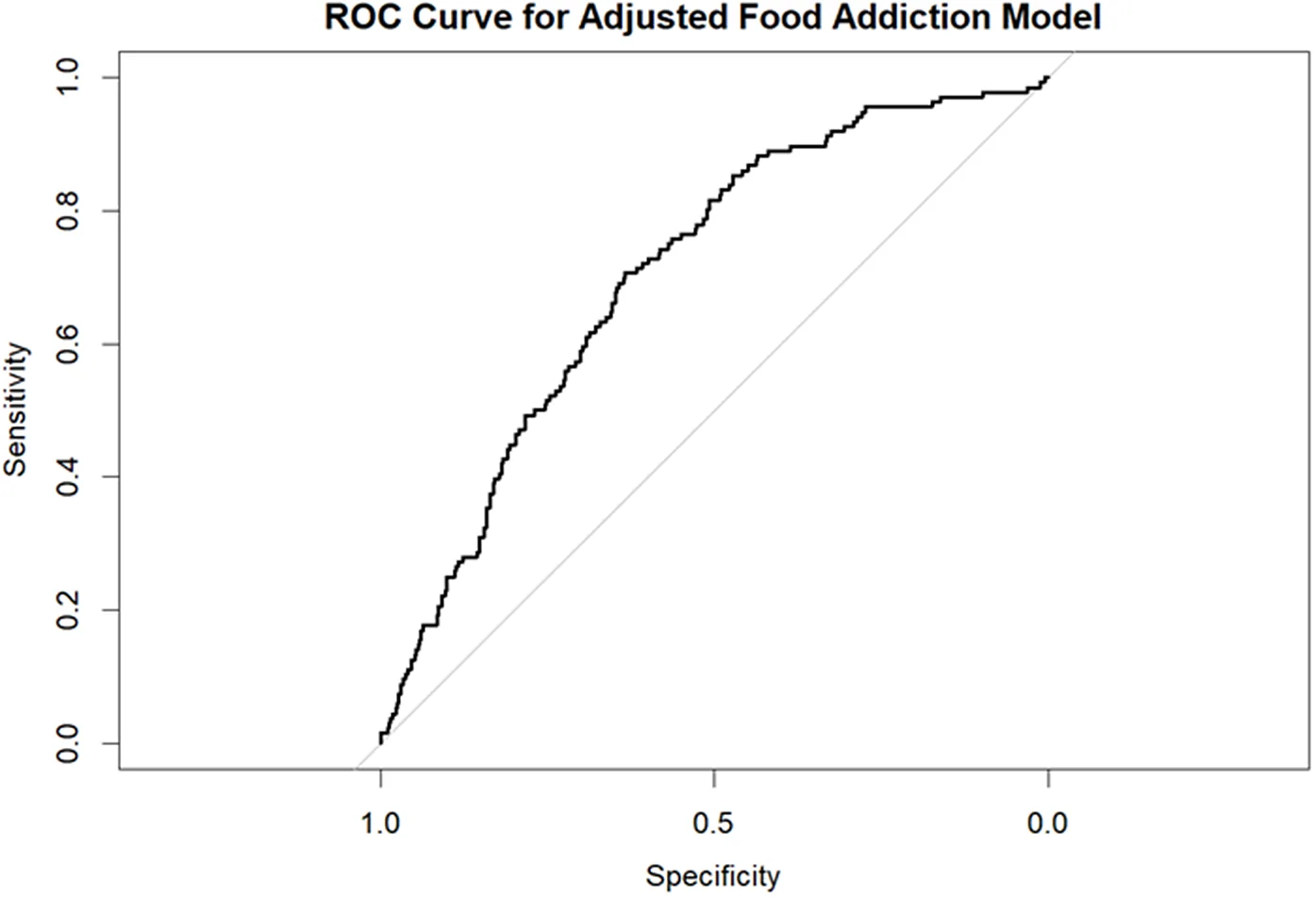
Receiver operating characteristic (ROC) curve for the adjusted food addiction model. The model shows acceptable discrimination with an AUC of 0.71.

Calibration performance is shown in Fig 5.

**Fig 5:**
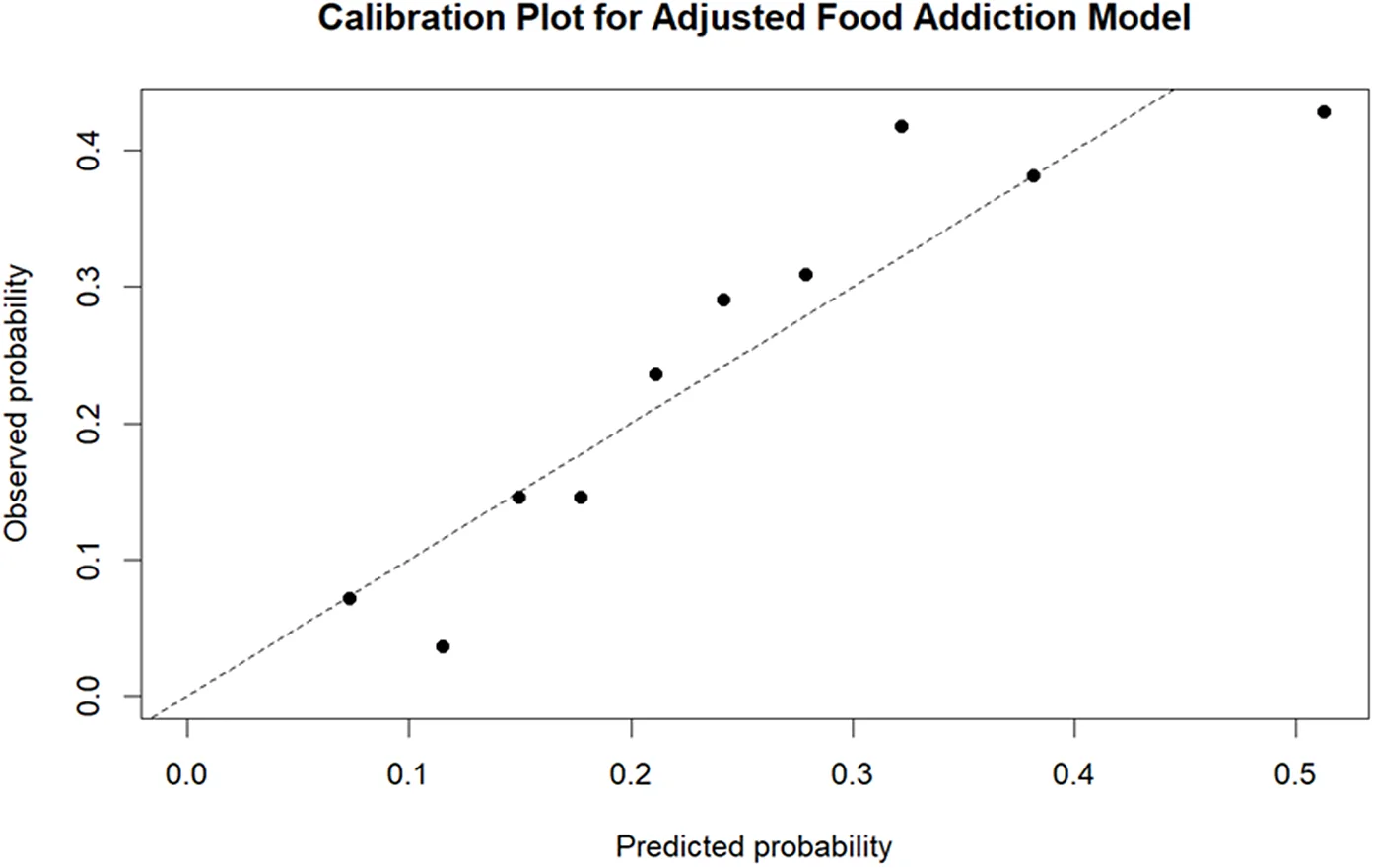
Calibration plot for the adjusted food addiction model. The plot demonstrates agreement between predicted and observed probabilities of food addiction across deciles of predicted risk.

### Internal consistency and construct validity

Internal consistency analysis demonstrated good reliability for the MSG composite scale, acceptable reliability for the Binge Eating Scale (BES), and low reliability for the Perceived Stress Scale–4 (PSS-4). The MSG composite, which consisted of 17 food items, showed strong internal coherence (Cronbach’s α = 0.835). The BES, composed of seven items, demonstrated acceptable internal consistency (α = 0.651). In contrast, the four-item PSS-4 showed lower reliability (α = 0.398), indicating limited internal consistency in this sample.

Spearman correlation analysis revealed a moderate positive association between total MSG intake and YFAS symptom count (ρ = 0.384). A similar moderate correlation was observed between BES scores and YFAS symptoms (ρ = 0.489), indicating substantial overlap between binge-eating severity and food addiction symptomatology. Weaker positive associations were found between MSG intake and BES scores (ρ = 0.205) and between MSG intake and perceived stress (ρ = 0.082). Correlations between perceived stress and both BES scores (ρ = 0.268) and YFAS symptoms (ρ = 0.257) were small but positive.

These reliability and correlation findings are summarized in Tables 4 and 5.

**Table 4:** Internal consistency (Cronbach’s alpha) of study measurement scales.

Scale	Number of items	Cronbach’s α
MSG composite	17	0.835
BES	7	0.651
PSS-4	4	0.398

**Table 5:** Spearman correlation matrix among primary study variables.

Variable	MSG total	BES total	YFAS symptoms	PSS total
MSG total	1.00	0.205	0.384	0.082
BES total	0.205	1.00	0.489	0.268
YFAS symptoms	0.384	0.489	1.00	0.257
PSS total	0.082	0.268	0.257	1.00

Overall, findings across multiple models consistently demonstrated a positive association between higher MSG intake and increased food addiction prevalence, symptom burden, and binge-eating severity.

## Discussion

This cross-sectional study explored the association between monosodium glutamate (MSG) intake and food addiction symptoms among university students in the West Bank, Palestine. Current research suggests that highly palatable foods, specifically those rich in flavor-enhancing additives, may interact with neural reward pathways associated with compulsive eating patterns [14]. Monosodium glutamate, a widely used flavor enhancer in processed and restaurant-prepared fast foods, is believed to increase palatability and potentially influence appetite regulation and food preferences [4]. Although MSG has been studied for consumption safety, its behavioral and dietary correlations remain less understood [15].

The observed association between high MSG intake and increased food addiction symptoms supports the hypothesis that frequent exposure to MSG-enhanced foods is associated with reward-driven eating behaviors. Experimental and theoretical models suggest that glutamate interacts with central reward pathways, increasing the desire for repeated consumption [16,17]. While MSG is considered safe from a toxicology perspective [15], the role it plays in modulating palatability and appetite regulation has been increasingly discussed in the context of obesogenic food environments [4].

Notably, the association persisted after adjusting BMI, stress, and dietary quality indicators, suggesting that MSG exposure may not simply reflect general unhealthy eating patterns but could represent a distinct dietary component linked to addictive-like eating behaviors. However, residual confounding cannot be excluded [18].

The Perceived Stress Scale-4 (PSS-4) demonstrated low internal consistency in this sample (Cronbach’s α = 0.398), suggesting limited reliability and potential attenuation of observed associations involving perceived stress. The Perceived Stress Scale is a brief instrument designed for rapid stress assessment [19]. Short scales are known to exhibit lower reliability coefficients compared to longer versions [20]. The limited number of items may decrease the scale’s ability to capture the multidimensional nature of stress [20]. Furthermore, cultural understanding of factors may have influenced item interpretation, potentially affecting internal consistency.

Given this low reliability, associations between perceived stress and other variables should be interpreted cautiously. Measurement error may attenuate the strength of observed associations, and stress may be underestimated as a confounder or predictor in regression models when measured with low-reliability instruments.

The logistic regression model demonstrated moderate discrimination (AUC ≈ 0.71), indicating moderate discriminatory performance. An AUC between 0.70 and 0.80 is generally interpreted as acceptable but not strong discrimination in clinical and behavioral research models [21]. This suggests that while MSG intake and included covariates contribute meaningfully to predicting food addiction status, they do not fully explain the variability in addictive-like eating behaviors.

Food addiction is widely understood to be a multifactorial phenomenon influenced by psychological traits, environmental exposures, and neurobiological vulnerabilities [15]. Unmeasured variables such as sleep quality, emotional regulation capacity, physical activity, socioeconomic constraints, and broader ultra-processed dietary patterns may enhance predictive accuracy in future models [22].

The Binge Eating Scale demonstrated acceptable internal consistency (Cronbach’s α = 0.651). The Binge Eating Scale has shown moderate-to-good reliability across diverse populations [23], though coefficients may vary depending on translation and cultural adaptation. Short behavioral instruments frequently yield moderate reliability values [24,25].

Despite this, the BES showed meaningful correlations with food addiction symptoms assessed using the Yale Food Addiction Scale [11], supporting convergent validity between binge eating and addictive-like eating behaviors.

### Weak correlation between MSG intake and perceived stress

The negligible correlation between MSG intake and perceived stress (ρ ≈ 0.08) suggests that MSG consumption in this study was not strongly driven by stress-related eating. Even though stress is frequently implicated in emotional eating behaviors [26,27], our findings indicate that the relationship between MSG exposure and food addiction symptoms is unlikely to be explained solely by stress-mediated mechanisms.

This relative independence strengthens the hypothesis that flavor-enhancers in food contribute to reward-driven eating behaviors through pathways different from those related to acute psychological stress.

### Non-significant demographic predictors

No significant differences were observed in age, BMI, stress levels, or gender distribution across MSG intake tertiles [28]. Baseline similarity reduces the likelihood that demographic confounding accounts for the observed associations [29,30]. Controlling for key covariates in observational research strengthens interpretability, although unmeasured confounding remains possible.

### Limitations

The cross-sectional design limits causal inference, because exposure and outcome were measured simultaneously, the temporal direction cannot be established. It remains unclear whether high MSG intake contributes to the development of addictive eating patterns or whether individuals predisposed to compulsive eating preferentially select highly palatable, MSG-rich foods. Longitudinal cohort studies and experimental feeding trials are required to clarify directionality and potential mechanisms.

Several relevant lifestyle factors were not assessed in this study, including physical activity, sleep quality, socioeconomic status, and overall dietary patterns such as ultra-processed food consumption. These variables may act as residual confounders and could influence both MSG intake and eating behaviors.

Additionally, individuals with a self-reported previous or current diagnosis of an eating disorder were excluded from participation. While this was intended to isolate addictive-like eating behaviors from clinically diagnosed pathology, it may have reduced the severity range of outcomes in the sample, potentially attenuating the observed associations between MSG intake and disordered eating behaviors.

### Public health implications

University students are frequently exposed to ultra-processed, flavor-enhanced foods due to time constraints, financial limitations, and campus food environments [31]. If MSG-rich dietary exposure contributes to reinforcement of addictive-like eating patterns, targeted nutritional education and environmental interventions aimed at reducing reliance on highly processed foods may be warranted. In addition, implementing community-based awareness programs and public health campaigns that promote healthier dietary choices and improve food literacy among students could help translate these findings into meaningful behavioral change at the population level. However, given the observational nature of this study, findings should be interpreted as hypothesis-generating rather than definitive evidence of causation.

## Conclusion

This study showed that higher MSG consumption among university students was associated with increased food addiction symptoms and binge eating behaviors. This relationship remained significant even after accounting for BMI, perceived stress, and dietary factors, suggesting that MSG consumption is associated with reward-driven eating patterns. Demographic factors such as age, gender, and academic year were not significant predictors, highlighting the primary role of dietary behaviors in compulsive eating. Additionally, MSG intake was not strongly related to stress, indicating that its effect on addictive-like eating behaviors may operate through mechanisms other than stress. The study also confirmed that binge eating scores were meaningfully related to food addiction symptoms, supporting the link between compulsive eating and addictive-like food behaviors. Overall, these findings provide clear evidence that frequent consumption of MSG-enhanced foods is associated with compulsive eating patterns among young adult students, though longitudinal research is needed to establish directionality.

## Supporting information

S1 File Study questionnaire. Bilingual (Arabic/English) self-administered questionnaire used for data collection, comprising five sections: demographic and anthropometric characteristics, MSG food frequency questionnaire (20 MSG-rich food items), Yale Food Addiction Scale 2.0 (YFAS 2.0), Binge Eating Scale (BES), and Perceived Stress Scale–4 (PSS-4).
(PDF)

S2 File Anonymized study dataset. Dataset generated and analyzed during the current study. All personally identifiable variables have been removed. The file contains all variables required to reproduce the statistical analyses presented.
(CSV)
