# Supplementary material for: Association between habitual MSG consumption and compulsive eating behaviors in university students: A cross-sectional study
Source: PLoS One. 2026 Nov 4;21(11):e0360433. doi: 10.1371/journal.pone.0360433 (PMC13734507; doi:10.1371/journal.pone.0360433)
Supplement: Supplementary file 1 [file pone.0360433.s001.pdf]

Association Between Habitual MSG Consumption and Compulsive Eating Behaviors in University Students: A Cross-Sectional Study

---

Section 1: Consent

1. Thank you for considering participation in this study. The purpose of this research is to understand how the frequent consumption of monosodium glutamate (MSG) in processed foods is associated with eating behaviors in young adults. Your participation is entirely voluntary, and all responses will be kept confidential and anonymous. No identifying information will be collected, and your data will be stored securely, accessible only to the research team. By proceeding with the questionnaire, you confirm that you have read and understood this information and agree to participate in the study.

Mark only one oval.

- I agree / أوافق

Section 2: Demographic Information

2. What is your age? / ما هو عمرك؟

Mark only one oval.

18 / 19 / 20 / 21 / 22 / 23 / 24 / 25 / 26 / 27 / 28 / 29 / 30 / older / أكبر

3. What is your gender? / ما هو جنسك؟

Mark only one oval.

- Male / ذكر

- Female / انثى

4. What College do you attend? / ما هي الكلية التي تدرس بها؟

Mark only one oval.

- College of Medicine and Health Sciences / كلية الطب والعلوم الصحية
- College of Pharmacy and Medical Science / كلية الصيدلة والعلوم الطبية
- College of Nursing / كلية التمريض
- College of Agriculture / كلية الزراعة
- College of Finance and Management / كلية المالية والإدارة
- College of Science and Technology / كلية العلوم والتكنولوجيا
- College of Education / كلية التربية
- College of Arts / كلية الآداب
- College of Law and Political Science / كلية القانون والعلوم السياسية
- College of Information Technology / كلية تكنولوجيا المعلومات
- College of Professions and Applied Sciences / كلية المهن والعلوم التطبيقية
- College of Islamic Studies / كلية الدراسات الإسلامية

5. What year of study are you in? / في أي سنة دراسية أنت؟

Mark only one oval.

- 1st year / السنة الأولى
- 2nd year / السنة الثانية
- 3rd year / السنة الثالثة
- 4th year / السنة الرابعة
- 5th year / السنة الخامسة
- 6th year or more / السنة السادسة أو أكثر

6. What is your height (in cm)? / ما هو طولك (سم)؟

7. What is your weight (in kg)? / ما هو وزنك (كجم)؟

Section 3: FFQ (Food Frequency Questionnaire)

8. Please indicate how often, on average, you have consumed each food over the past 12 months. / يرجى الإشارة إلى عدد المرات التي استهلكت فيها كل نوع من الأطعمة في المتوسط خلال الأشهر الـ 12 الماضية

Mark only one oval per row.

Food Item	Never / أبداً	1-3 times per month / 1-3 مرات في الشهر	1-2 times per week / 1-2 مرات في الأسبوع	3-4 times per week / 3-4 مرات في الأسبوع	5 or more times per week / 5 مرات أو أكثر في الأسبوع
Instant Noodles / الرامن والإندومي	○	○	○	○	○
Potato Chips / Crisps / الشبس	○	○	○	○	○
Flavored Crackers (e.g., Cheez-Its, Goldfish) / البسكويت المنكّه (مثل	○	○	○	○	○

بسكويت الجبن)					
Fast Food Burgers & Fries / برجر وبطاطس سريعة	○	○	○	○	○
Fried Chicken (from fast-food or takeout) / دجاج مقلي (من الوجبات السريعة أو الوجبات الجاهزة)	○	○	○	○	○
Soy Sauce / Teriyaki Sauce /	○	○	○	○	○

صلصة الصويا / صلصة ترياكي					
Salad Dressings / صلصات السلطة	○	○	○	○	○
Bouillon Cubes / مكعبات المرق	○	○	○	○	○
Seasoning Packets / أكياس التوابل	○	○	○	○	○
Pretzels / البسكويت المالح (بريتزل)	○	○	○	○	○
Chinese Takeout	○	○	○	○	○

الوجبات / الصينية الجاهزة					
Condiments / الصلصات (كاتشب، مايونيز)	○	○	○	○	○
Pre-Packaged Deli Meats (e.g., sliced turkey, mortadella) / اللحوم الباردة المغلفة (مثل الديك الرومي المقطع، المرتديلا)	○	○	○	○	○
Cheese-flavored	○	○	○	○	○

popcorn / الفشار بنكهة الجبن					
Pizza / بيتزا	○	○	○	○	○
Nacho cheese sauce / صلصة الجبن الناتشو	○	○	○	○	○
Tomato paste / معجون الطماطم	○	○	○	○	○

Section 4: YFAS (Yale Food Addiction Scale)

9. The following questions are about your relationship with food. Please think about the past 12 months when answering. / الأسئلة التالية تتعلق بعلاقتك بالطعام. يُرجى التفكير في الأشهر الإثني عشر الماضية عند الإجابة

Mark only one oval per row.

Statement	Never / أبداً	Once a month or less / مرة واحدة في الشهر أو أقل	2-4 times a month / 2-4 مرات في الشهر	2-3 times a week / 2-3 مرات في الأسبوع	4+ times a week / 4+ مرات في الأسبوع
1. I ate certain foods so often or in such large amounts that I spent a lot of my time eating or feeling sluggish from overeating. / كنت أتناول بعض الأطعمة بشكل متكرر أو بكميات كبيرة لدرجة أنني قضيت	○	○	○	○	○

الكثير من وقتي في الأكل أو أشعر بالخمول بسبب الإفراط في تناول الطعام					
2. I had such strong urges to eat certain foods that I couldn't think of anything else. / كنت لدي رغبة قوية في تناول أطعمة معينة لدرجة أنني لا أستطيع	○	○	○	○	○

التفكير في أي شيء آخر					
3. I avoided professional, social or recreational activities because I feared I would overeat there. / تجنبت الأنشطة المهنية أو الاجتماعية أو الترفيهية لأنني خشيت أن أفرط في تناول الطعام هناك	○	○	○	○	○

4. I had withdrawal symptoms like agitation, anxiety or physical effects when I cut down on certain foods (not including caffeine). / عانيت من أعراض انسحابية مثل الهياج أو القلق أو تأثيرات جسدية عندما قللت من تناول أطعمة	○	○	○	○	○

معينة (لا يشمل الكافيين)					
5. I needed to eat more and more of the same foods to get the feeling I wanted, like reduced negative emotions or pleasure. / احتجت إلى تناول المزيد والمزيد من نفس الأطعمة للحصول على الشعور الذي أريده، مثل تقليل	○	○	○	○	○

المشاعر السلبية أو المتعة					
6. I kept eating certain foods even though I knew they were causing significant emotional or physical problems for me (e.g., feeling sad, guilty, bloated, or ill). / استمريت في	○	○	○	○	○

تناول أطعمة معينة على الرغم من أنني كنت أعلم أنها تسبب لي مشاكل عاطفية أو جسدية كبيرة (مثل الشعور بالحزن أو الذنب أو الانتفاخ أو المرض)					
7. I found that I needed to eat more of certain foods to get the same feeling I used to get from smaller	○	○	○	○	○

amounts. / وجدت أنني بحاجة إلى تناول المزيد من أطعمة معينة للحصول على نفس الشعور الذي اعتدت الحصول عليه من كميات أقل					
8. I tried to cut down on or stop eating certain foods but I wasn't successful. / حاولت تقليل أو التوقف عن تناول أطعمة	○	○	○	○	○

معينة لكنني لم أكن ناجحاً					
9. I continued to eat the same types of food or the same amount of food even though I wanted to cut down or stop. / استمريت في تناول نفس الأنواع من الطعام أو نفس الكمية من الطعام على الرغم من أنني أردت التقليل أو التوقف	○	○	○	○	○

10. I had such a strong desire for certain foods that I couldn't keep from eating them when I wanted to. / كانت لدي رغبة قوية لدرجة أنني لم أستطع الامتناع عن تناول أطعمة معينة عندما أريد ذلك	○	○	○	○	○

11. I found that when I started eating certain foods, I ended up eating much more than I had planned. / وجدت أنه عندما أبدأ في تناول أطعمة معينة، أنتهي بتناول كمية أكبر بكثير مما خططت له	○	○	○	○	○

10. The problems I had with these foods caused me significant distress. / لقد سببت لي المشاكل التي واجهتها مع هذه الأطعمة ضائقة كبيرة

Mark only one oval.

- Yes / نعم

- No / لا

11. The problems I had with these foods interfered with my ability to work, go to school, or socialize. / لقد أثرت المشاكل التي واجهتها مع هذه الأطعمة على قدرتي على العمل أو الذهاب إلى المدرسة أو التواصل الاجتماعي

Mark only one oval.

- Yes / نعم
- No / لا

Section 5: BES (Binge Eating Scale)

12. Please select the statement you most relate to. / الرجاء تحديد العبارة التي تتعلق بها أكثر

Mark only one oval.

- I don't feel any guilt or self-hatred after I overeat. / لا أشعر بالذنب أو كراهية الذات بعد الإفراط في تناول الطعام.
- Sometimes I feel guilty or down after overeating. / أحياناً أشعر بالذنب أو الإحباط بعد الإفراط في الأكل.
- I feel so guilty or disgusted with myself after overeating that I can't stop thinking about it. / أشعر بالذنب أو الاشمئزاز من نفسي لدرجة أنني لا أستطيع التوقف عن التفكير في الأمر.
- I feel incredibly guilty, depressed, and distressed after overeating. / أشعر بالذنب الشديد والاكتئاب والضيق بعد الإفراط في الأكل.

13. Please select the statement you most relate to. / الرجاء تحديد العبارة التي تتعلق بها أكثر

Mark only one oval.

- I can't seem to stop eating once I start. / لا أستطيع التوقف عن الأكل بمجرد أن أبدأ.
- I have difficulty stopping eating once I start, but I can usually stop when I want to. / أواجه صعوبة في التوقف عن الأكل بمجرد أن أبدأ، لكنني عادةً أستطيع التوقف عندما أرغب في ذلك.
- I feel like I have control over my eating; I can stop when I want to. / أشعر أن لدي سيطرة على أكلي؛ أستطيع التوقف عندما أرغب.

14. Please select the statement you most relate to. / الرجاء تحديد العبارة التي تتعلق بها أكثر

Mark only one oval.

- I eat normally; no faster or slower than most people. / أكلي طبيعي؛ لا أسرع أو أبطأ من معظم الناس.
- I tend to eat slightly faster than other people. / أميل إلى الأكل بشكل أسرع قليلاً من الآخرين.
- I eat much faster than other people, often gulping my food down. / آكل بشكل أسرع بكثير من الآخرين، وغالبًا ما أبتلع طعامي بسرعة.

15. Please select the statement you most relate to. / الرجاء تحديد العبارة التي تتعلق بها أكثر

Mark only one oval.

- I don't eat any more than usual when I'm not hungry. / لا آكل أكثر من المعتاد عندما لست جائعاً.
- Sometimes I eat more when I'm not hungry. / أحياناً آكل أكثر عندما لست جائعاً.
- I often eat large amounts of food even when I'm not physically hungry. / غالباً ما آكل كميات كبيرة من الطعام حتى عندما لست جائعاً جسدياً.

16. Please select the statement you most relate to. / الرجاء تحديد العبارة التي تتعلق بها أكثر

Mark only one oval.

- I don't eat in secret. / لا آكل في الخفاء.
- Sometimes I prefer to eat alone so others don't see how much I'm consuming. / أحياناً أفضل أن أتناول الطعام بمفردي حتى لا يرى الآخرون كمية الطعام التي أستهلكها.
- I often eat in secret because I'm embarrassed about how much I eat. / غالباً ما آكل في الخفاء لأنني أشعر بالحرج من الكمية التي أستهلكها.

17. Please select the statement you most relate to. / الرجاء تحديد العبارة التي تتعلق بها أكثر

Mark only one oval.

- I never feel driven to eat for emotional reasons. / لا أشعر أبدًا بدافع قوي للأكل لأسباب عاطفية
- Sometimes I feel driven to eat when I'm stressed or upset. / أحيانًا أشعر بدافع قوي للأكل عندما أكون متوترًا أو منزعجًا
- I often feel a strong, uncontrollable drive to eat, especially when emotional. / غالبًا ما أشعر بدافع قوي ولا يمكن السيطرة عليه للأكل، خاصة عندما أكون في حالة عاطفية.

18. Please select the statement you most relate to. / الرجاء تحديد العبارة التي تتعلق بها أكثر

Mark only one oval.

- I rarely think about food outside of meal times. / نادراً ما أفكر في الطعام خارج أوقات الوجبات.

- I sometimes find myself thinking about food between meals. / أحيانًا أجد نفسي أفكر في الطعام بين الوجبات.
- I am constantly preoccupied with thoughts about food and eating. / أنا منشغل دائمًا بأفكار حول الطعام والأكل.

Section 6: PSS-4 (Perceived Stress Scale – 4 item)

19. For each question, please indicate how often you felt or thought a certain way. / بالنسبة لكل سؤال، يرجى الإشارة إلى عدد المرات التي شعرت أو فكرت فيها بطريقة معينة

Mark only one oval per row.

Question	Never / أبداً	Almost never / تقريباً أبداً	Sometimes / أحياناً	Fairly often / غالباً إلى حد ما	Very often / كثيراً جداً
In the last month, how often have you felt that you were unable to control the important things in your life? / في الشهر الماضي، كم مرة شعرت أنك غير قادر على التحكم في	○	○	○	○	○

الأمور المهمة في حياتك					
In the last month, how often have you felt confident about your ability to handle your personal problems? / في الشهر الماضي، كم مرة شعرت بالثقة في قدرتك على التعامل مع مشاكلك الشخصية	○	○	○	○	○
In the last month, how often have you felt that things were going your way? / في الشهر الماضي، كم مرة شعرت أن الأمور تسير في طريقك	○	○	○	○	○

In the last month, how often have you felt difficulties were piling up so high that you could not overcome them? / في الشهر الماضي، كم مرة شعرت أن الصعوبات تتراكم لدرجة أنك لا تستطيع التغلب عليها	○	○	○	○	○

Final Questions

20. Have you ever been diagnosed with an eating disorder (e.g., anorexia nervosa, bulimia nervosa, binge eating disorder) by a medical professional? / هل سبق أن شخصت باضطراب في الأكل (مثل فقدان الشهية العصبي، الشره العصبي، اضطراب الأكل الشراهة) من قبل متخصص طبي؟

Mark only one oval.

- Yes / نعم
- No / لا

21. Is there anything else about your eating habits or this survey you would like to share? / هل هناك أي شيء آخر حول عاداتك الغذائية أو هذا الاستبيان الذي ترغب في مشاركته؟

End of questionnaire. Thank you for your participation. / نهاية الاستبيان. شكرا لمشاركتك.
