# Supplementary material for: Association between habitual MSG consumption and compulsive eating behaviors in university students: A cross-sectional study
Source: PLoS One. 2026 Nov 4;21(11):e0360433. doi: 10.1371/journal.pone.0360433 (PMC13734507; doi:10.1371/journal.pone.0360433)
Supplement: Supplementary file 2 [file pone.0360433.s003.docx]

**Review PONE-D-26-22168**

Dear authors congratulations on your paper, I enjoyed reading it. It has enough quality to be published. The manuscript is well organized and written clearly enough.

Nevertheless, I have some considerations about your paper that can improve their quality, considering the following points:

In methodology I recommended to properly include the citation in the questionnaires used: YFAS 2.0, PSS-4 and BES.

The ethical approval is not really clear, because normally in these documents all the contain in it was typed not handwritten.

Tables must have a header explained they, as you presented the Figures.

This paragraph is typed with different letter “The logistic regression model demonstrated moderate discrimination (AUC ≈ 0.71), indicating moderate discriminatory performance. An AUC between 0.70 and 0.80 is generally interpreted as acceptable but not strong discrimination in clinical and behavioral research models [17]. This suggests that while MSG intake and included covariates contribute meaningfully to predicting food addiction status, they do not fully explain the variability in addictive-like eating behaviors.”

I recommended to review these three references:

[2] Monosodium glutamate: Review on clinical reports. International Journal of Food Properties 2017. https://doi.org/10.1080//10942912.2017.1295260.

[10] Reconsideration of the health effects of monosodium glutamate: from bench to bedside evidence.Journal of Environmental Science and Health, Part C 2025.

<https://doi.org/10.1080//26896583.2024.2415202>.

[18] Stress and eating behaviours in healthy adults: a systematic review and meta-analysis. Health Psychology Review 2022. <https://doi.org/10.1080//17437199.2021.1923406>.

Also I recommended the authors to include more recently published bibliography and more articles into the references
