# Supplementary material for: Association between habitual MSG consumption and compulsive eating behaviors in university students: A cross-sectional study
Source: PLoS One. 2026 Nov 4;21(11):e0360433. doi: 10.1371/journal.pone.0360433 (PMC13734507; doi:10.1371/journal.pone.0360433)
Supplement: Peer review history

# Peer review: Decision Letter 0

**DOI:** 10.1371/journal.pone.0360433.r001

**Authors:** Junzheng Yang

28 Jun 2026

Dear Dr. Aljabry,

Thank you for submitting your manuscript to PLOS ONE. After careful consideration, we feel that it has merit but does not fully meet PLOS ONE’s publication criteria as it currently stands. Therefore, we invite you to submit a revised version of the manuscript that addresses the points raised during the review process.

Please submit your revised manuscript by Jul 28 2026 11:59PM. If you will need more time than this to complete your revisions, please reply to this message or contact the journal office at plosone@plos.org. When you're ready to submit your revision, log on to https://www.editorialmanager.com/pone/ and select the 'Submissions Needing Revision' folder to locate your manuscript file.

- A letter that responds to each point raised by the academic editor and reviewer(s). You should upload this letter as a separate file labeled 'Response to Reviewers'.
- A marked-up copy of your manuscript that highlights changes made to the original version. You should upload this as a separate file labeled 'Revised Manuscript with Track Changes'.
- An unmarked version of your revised paper without tracked changes. You should upload this as a separate file labeled 'Manuscript'.

If you would like to make changes to your financial disclosure, please include your updated statement in your cover letter. Guidelines for resubmitting your figure files are available below the reviewer comments at the end of this letter.

If applicable, we recommend that you deposit your laboratory protocols in protocols.io to enhance the reproducibility of your results. Protocols.io assigns your protocol its own identifier (DOI) so that it can be cited independently in the future. For instructions see: https://journals.plos.org/plosone/s/submission-guidelines#loc-laboratory-protocols. Additionally, PLOS ONE offers an option for publishing peer-reviewed Lab Protocol articles, which describe protocols hosted on protocols.io. Read more information on sharing protocols at https://plos.org/protocols?utm_medium=editorial-email&utm_source=authorletters&utm_campaign=protocols.

As the corresponding author, your ORCID iD is verified in the submission system and will appear in the published article. PLOS supports the use of ORCID, and we encourage all coauthors to register for an ORCID iD and use it as well. Please encourage your coauthors to verify their ORCID iD within the submission system before final acceptance, as unverified ORCID iDs will not appear in the published article. *Only*  the individual author can complete the verification step; PLOS staff *cannot*  verify ORCID iDs on behalf of authors.

We look forward to receiving your revised manuscript.

Kind regards,

Junzheng Yang

Academic Editor

PLOS One

Journal Requirements:

When submitting your revision, we need you to address these additional requirements.

1. Please ensure that your manuscript meets PLOS ONE's style requirements, including those for file naming. The PLOS ONE style templates can be found at

https://journals.plos.org/plosone/s/file?id=wjVg/PLOSOne_formatting_sample_main_body.pdf and https://journals.plos.org/plosone/s/file?id=ba62/PLOSOne_formatting_sample_title_authors_affiliations.pdf

2. Your ethics statement should only appear in the Methods section of your manuscript. If your ethics statement is written in any section besides the Methods, please delete it from any other section.

3. Please provide a complete Data Availability Statement in the submission form, ensuring you include all necessary access information or a reason for why you are unable to make your data freely accessible. If your research concerns only data provided within your submission, please write "All data are in the manuscript and/or supporting information files" as your Data Availability Statement.

4. Please update your submission to use the PLOS LaTeX template. The template and more information on our requirements for LaTeX submissions can be found at http://journals.plos.org/plosone/s/latex.

5. We note you have included a table to which you do not refer in the text of your manuscript. Please ensure that you refer to Table 1, 2 in your text; if accepted, production will need this reference to link the reader to the Table.

6. Please include captions for your Supporting Information files at the end of your manuscript, and update any in-text citations to match accordingly. Please see our Supporting Information guidelines for more information: http://journals.plos.org/plosone/s/supporting-information.

7. We note that there is identifying data in the Supporting Information file <MSG Dataset 1.csv>. Due to the inclusion of these potentially identifying data, we have removed this file from your file inventory. Prior to sharing human research participant data, authors should consult with an ethics committee to ensure data are shared in accordance with participant consent and all applicable local laws.

Data sharing should never compromise participant privacy. It is therefore not appropriate to publicly share personally identifiable data on human research participants. The following are examples of data that should not be shared:

-Name, initials, physical address

-Ages more specific than whole numbers

-Internet protocol (IP) address

-Specific dates (birth dates, death dates, examination dates, etc.)

-Contact information such as phone number or email address

-Location data

-ID numbers that seem specific (long numbers, include initials, titled “Hospital ID”) rather than random (small numbers in numerical order)

Data that are not directly identifying may also be inappropriate to share, as in combination they can become identifying. For example, data collected from a small group of participants, vulnerable populations, or private groups should not be shared if they involve indirect identifiers (such as sex, ethnicity, location, etc.) that may risk the identification of study participants.

Additional guidance on preparing raw data for publication can be found in our Data Policy (https://journals.plos.org/plosone/s/data-availability#loc-human-research-participant-data-and-other-sensitive-data) and in the following article: http://www.bmj.com/content/340/bmj.c181.long.

Please remove or anonymize all personal information (<specific identifying information in file to be removed>), ensure that the data shared are in accordance with participant consent, and re-upload a fully anonymized data set. Please note that spreadsheet columns with personal information must be removed and not hidden as all hidden columns will appear in the published file.

8. If the reviewer comments include a recommendation to cite specific previously published works, please review and evaluate these publications to determine whether they are relevant and should be cited. There is no requirement to cite these works unless the editor has indicated otherwise.

9. Please review your reference list to ensure that it is complete and correct. If you have cited papers that have been retracted, please include the rationale for doing so in the manuscript text, or remove these references and replace them with relevant current references. Any changes to the reference list should be mentioned in the rebuttal letter that accompanies your revised manuscript. If you need to cite a retracted article, indicate the article’s retracted status in the References list and also include a citation and full reference for the retraction notice.

[Note: HTML markup is below. Please do not edit.]

Reviewers' comments:

Reviewer's Responses to Questions

**Comments to the Author**

1. Is the manuscript technically sound, and do the data support the conclusions?

Reviewer #1: Yes

Reviewer #2: Yes

**********

2. Has the statistical analysis been performed appropriately and rigorously?

Reviewer #1: N/A

Reviewer #2: Yes

**********

3. Have the authors made all data underlying the findings in their manuscript fully available?

The PLOS Data policy

Reviewer #1: Yes

Reviewer #2: Yes

**********

4. Is the manuscript presented in an intelligible fashion and written in standard English?

Reviewer #1: Yes

Reviewer #2: Yes

**********

Reviewer #1: Dear authors, congratulations on your paper, congratulations to all of you. You presented an interesting study. The manuscript is well organized and written clearly enough. I attached the letter with my comments. After your revision, the paper should be published

Reviewer #2: 1. Were the people who received the questionnaire initially also distributed among their contacts? or through any other way snowball sampling was done?

2.To mention how the language validation was done for the bi-lingual questionnaire.

3 Was the expert review done by delphi method or any other method?.

4. In page 19, a different font has been used.

**********

what does this mean?). If published, this will include your full peer review and any attached files.

If you choose “no”, your identity will remain anonymous but your review may still be made public.

**Do you want your identity to be public for this peer review?**  For information about this choice, including consent withdrawal, please see our Privacy Policy

Reviewer #1: No

Reviewer #2: **Yes:**  Dr Satabdi Mitra

**********

[NOTE: If reviewer comments were submitted as an attachment file, they will be attached to this email and accessible via the submission site. Please log into your account, locate the manuscript record, and check for the action link "View Attachments". If this link does not appear, there are no attachment files.]

To ensure your figures meet our technical requirements, please review our figure guidelines: https://journals.plos.org/plosone/s/figures

You may also use PLOS’s free figure tool, NAAS, to help you prepare publication quality figures: https://journals.plos.org/plosone/s/figures#loc-tools-for-figure-preparation.

NAAS will assess whether your figures meet our technical requirements by comparing each figure against our figure specifications.

Attachment Submitted filename: Review PONE.docx

# Author comment: Author response to Decision Letter 1

**DOI:** 10.1371/journal.pone.0360433.r002

29 Jun 2026

Dear Editor,

We sincerely thank the editorial team and both reviewers for their careful reading of our manuscript and their constructive feedback. We have revised the manuscript in full accordance with all editor and reviewer comments. A point-by-point response is provided below, with each change described and its location in the revised manuscript indicated. All changes are also highlighted in the tracked-changes version of the manuscript submitted alongside this letter.

RESPONSE TO EDITOR COMMENTS

Editor Comment 1 (PLOS ONE Style Requirements):

The revised manuscript has been formatted in accordance with PLOS ONE style guidelines. We have reviewed the style templates provided and ensured that all sections, headings, and text elements conform to the required format.

Editor Comment 2 (Ethics Statement Placement):

The ethics statement appears exclusively in the Methods section of the revised manuscript and has been removed from all other sections.

Editor Comment 3 (Data Availability Statement):

The Data Availability Statement has been updated in the submission form. The anonymized dataset has been re-uploaded as Supporting Information S3 File, and the statement now reads: "The anonymized dataset supporting the findings of this study is available as Supporting Information (S3 File). All personally identifiable information has been removed in accordance with participant consent and applicable data protection guidelines."

Editor Comment 4 (LaTeX Template):

The manuscript was prepared using Microsoft Word and does not use LaTeX formatting. No action is required for this point.

Editor Comment 5 (In-Text Table References):

We confirm that all five tables are explicitly cited in the manuscript text prior to their appearance. Table 1 is cited at the end of the Participant Characteristics sub-section; Table 2 at the end of Baseline Characteristics by MSG Tertile; Table 3 in the Association between MSG Intake and Food Addiction Diagnosis sub-section; and Tables 4 and 5 in the Internal Consistency and Construct Validity sub-section.

Editor Comment 6 (Supporting Information Captions):

Captions for all Supporting Information files have been added at the end of the manuscript, after the References section, as follows: S1 File (study questionnaire), S2 File (IRB ethical approval), and S3 File (anonymized dataset). In-text citations to these files have been updated accordingly in the Methods section.

Editor Comment 7 (Dataset Anonymization):

We thank the editor for this important notice. The original dataset has been fully anonymized and re-uploaded as S3 File. Specifically, the following were removed or modified:

• The consent/intro column (a Google Forms artifact with no analytical value) was removed.

• The college/faculty variable was removed, as two colleges had group sizes of 1 and 3 participants respectively, creating re-identification risk when combined with other variables.

• The open-text free-response column was removed, as it contained personal health disclosures from participants.

• Four participants with ages above 25 (each unique in the dataset) were recoded as ’25+’ to prevent singling out. All 47 substantive analytical variables are fully preserved.

Editor Comment 8 (Reviewer-Recommended Citations):

We have reviewed the references recommended by Reviewer 1 and incorporated those deemed relevant into the revised manuscript, as detailed in our response to Reviewer 1 below.

Editor Comment 9 (Reference List Corrections):

Three references contained malformed DOIs (double-slash errors: 10.1080// instead of 10.1080/). These have been corrected, and previously incomplete citations have been updated with full author names and bibliographic details.

RESPONSE TO REVIEWER 1

We thank Reviewer 1 for the positive overall assessment and for the specific and constructive suggestions that have meaningfully strengthened the manuscript.

Comment 1 (Validation citations for YFAS 2.0, BES, and PSS-4):

We agree that the original validation references for each instrument should be cited at first mention in the Methods section. We have added the citations into the text.

Comment 2 (Ethics approval clarity):

We acknowledge the reviewer’s observation that the IRB document appears handwritten. We wish to clarify that Hebron University College of Medicine’s Institutional Review Board issues approvals in this format as standard institutional practice. The approval number (ER.CM.68.2025), institutional name, and study title are clearly legible in the submitted document. The study was conducted in full accordance with the Declaration of Helsinki, and informed consent was obtained from all participants electronically at the start of the questionnaire.

Comment 3 (Table titles):

All five table titles have been updated to be fully descriptive, consistent with the approach used for figure captions throughout the manuscript.

Comment 4 (Font inconsistency in Discussion):

The paragraph discussing the AUC value (beginning “The logistic regression model demonstrated moderate discrimination…”) has been reformatted to match the font, size, and spacing of the surrounding text throughout the Discussion section.

Comment 5 (Reference corrections):

Addressed under Editor Comment 9 above.

RESPONSE TO REVIEWER 2

We thank Reviewer 2 for the methodological questions, which have helped us improve the clarity and rigor of the Methods section.

Comment 1 (Snowball sampling):

We have clarified the snowball sampling procedure in the Methods section. The questionnaire link was initially shared via social media platforms (WhatsApp, Instagram, and university student group pages). Participants were then encouraged to forward the link to fellow students in their networks, extending reach across multiple universities and academic years.

Comment 2 (Bilingual questionnaire validation):

We have added a description of the language validation process in the Methods section. The questionnaire was developed in English and translated into Arabic using a forward-backward translation procedure: one bilingual researcher translated the instrument into Arabic, and an independent bilingual translator back-translated it into English. Discrepancies were reviewed and resolved by consensus among the research team to ensure semantic equivalence across both language versions.

Comment 3 (Expert review method):

We have clarified this in the Methods section. The questionnaire was reviewed by a panel of three independent experts using a consensus-based approach. Each expert independently assessed the questionnaire for clarity, relevance, and comprehensiveness and provided feedback, which was subsequently discussed and incorporated by the research team.

Comment 4 (Font on page 19):

Addressed under Reviewer 1 Comment 4 above. The formatting inconsistency has been corrected throughout.

We believe the revised manuscript is substantially strengthened by these changes and hope it now meets the standards for publication in PLOS ONE. We remain available to address any further questions from the editorial team.

Sincerely,

Lina K. Al Jabry, and co-authors

College of Medicine, Hebron University, Hebron, Palestine

lina.aljabry06@gmail.com

# Peer review: Decision Letter 1

**DOI:** 10.1371/journal.pone.0360433.r003

**Authors:** Junzheng Yang

4 Sep 2026

Dear Dr. Aljabry,

Thank you for submitting your manuscript to PLOS One. After careful consideration, we feel that it has merit but does not fully meet PLOS One’s publication criteria as it currently stands. Therefore, we invite you to submit a revised version of the manuscript that addresses the points raised during the review process.

Please submit your revised manuscript by Oct 04 2026 11:59PM. If you will need more time than this to complete your revisions, please reply to this message or contact the journal office at plosone@plos.org. When you're ready to submit your revision, log on to https://www.editorialmanager.com/pone/ and select the 'Submissions Needing Revision' folder to locate your manuscript file.

- A letter that responds to each point raised by the academic editor and reviewer(s). You should upload this letter as a separate file labeled 'Response to Reviewers'.
- A marked-up copy of your manuscript that highlights changes made to the original version. You should upload this as a separate file labeled 'Revised Manuscript with Track Changes'.
- An unmarked version of your revised paper without tracked changes. You should upload this as a separate file labeled 'Manuscript'.

If you would like to make changes to your financial disclosure, please include your updated statement in your cover letter. Guidelines for resubmitting your figure files are available below the reviewer comments at the end of this letter.

If applicable, we recommend that you deposit your laboratory protocols in protocols.io to enhance the reproducibility of your results. Protocols.io assigns your protocol its own identifier (DOI) so that it can be cited independently in the future. For instructions see: https://journals.plos.org/plosone/s/submission-guidelines#loc-laboratory-protocols. Additionally, PLOS One offers an option for publishing peer-reviewed Lab Protocol articles, which describe protocols hosted on protocols.io. Read more information on sharing protocols at https://plos.org/protocols?utm_medium=editorial-email&utm_source=authorletters&utm_campaign=protocols.

As the corresponding author, your ORCID iD is verified in the submission system and will appear in the published article. PLOS supports the use of ORCID, and we encourage all coauthors to register for an ORCID iD and use it as well. Please encourage your coauthors to verify their ORCID iD within the submission system before final acceptance, as unverified ORCID iDs will not appear in the published article. *Only*  the individual author can complete the verification step; PLOS staff *cannot*  verify ORCID iDs on behalf of authors.

We look forward to receiving your revised manuscript.

Kind regards,

Junzheng Yang

Academic Editor

PLOS One

Journal Requirements:

1. If the reviewer comments include a recommendation to cite specific previously published works, please review and evaluate these publications to determine whether they are relevant and should be cited. There is no requirement to cite these works unless the editor has indicated otherwise.

2. Please review your reference list to ensure that it is complete and correct. If you have cited papers that have been retracted, please include the rationale for doing so in the manuscript text, or remove these references and replace them with relevant current references. Any changes to the reference list should be mentioned in the rebuttal letter that accompanies your revised manuscript. If you need to cite a retracted article, indicate the article’s retracted status in the References list and also include a citation and full reference for the retraction notice.

[Note: HTML markup is below. Please do not edit.]

Reviewer's Responses to Questions

**Comments to the Author**

Reviewer #1: All comments have been addressed

Reviewer #3: All comments have been addressed

**********

2. Is the manuscript technically sound, and do the data support the conclusions?

Reviewer #1: Yes

Reviewer #3: Yes

**********

3. Has the statistical analysis been performed appropriately and rigorously?

Reviewer #1: Yes

Reviewer #3: Yes

**********

4. Have the authors made all data underlying the findings in their manuscript fully available?

The PLOS Data policy

Reviewer #1: (No Response)

Reviewer #3: Yes

**********

5. Is the manuscript presented in an intelligible fashion and written in standard English?

Reviewer #1: (No Response)

Reviewer #3: Yes

**********

Reviewer #1: Dear Editor, In my opinion this paper is available for publication, because the authors have met all the recomendations..

Reviewer #3: This study presents a novel and potentially significant contribution to the literature on food addiction and dietary factors. The investigation of MSG, a common but often overlooked food additive, as a correlate of addictive-like eating behaviors in a Middle Eastern population is a clear strength. The use of validated instruments (YFAS, BES) and a rigorous analytical approach (including sensitivity analyses and model diagnostics) are commendable. However, some minor revisions are needed to improve its overall quality.

1. In abstract, the final sentence, "...suggesting frequent consumption of flavor-enhanced foods *may contribute* to reward-driven eating behaviors," uses causal language ("contribute to"). In an abstract for a cross-sectional study, this should be softened. I recommend: "...suggesting frequent consumption of flavor-enhanced foods is a correlate of reward-driven eating behaviors, warranting further investigation."

2. The abbreviation "AUC" is used without being defined in the abstract.

3. In method section: Exclusion Criteria: "Self-reported previous or current diagnosis of an eating disorder" is a valid exclusion. However, by excluding these individuals, you may be reducing your sample size and the severity of the outcome, potentially attenuating your findings. This should be discussed as a limitation.

4. Pilot Study: You mention a pilot study was conducted to "evaluate feasibility and reliability." The results of this pilot (e.g., the initial Cronbach's alpha values) are not reported. If the pilot led to modifications, what were they?

5. In discussion section, there is over-interpretation of "causality": The discussion begins by framing the question as "explored the association," which is correct. However, later statements like "frequent exposure to MSG-enhanced foods *may reinforce* reward-driven eating behaviors" ("reinforce" implies causality) and the conclusion "...may *contribute* to compulsive eating patterns" are too causal. You must consistently emphasize the cross-sectional, associative nature of your findings throughout the discussion. The "Public Health Implications" section is appropriately cautious, ending with "hypothesis-generating rather than definitive evidence of causation," but this caution should permeate the entire discussion.

6. In the reference list, you may consider adding the following related reference, which is closely relevant to the topic of this manuscript: Assessing nutritional adequacy ratios in women with and without binge eating disorder: a comprehensive evaluation.

**********

what does this mean?). If published, this will include your full peer review and any attached files.

If you choose “no”, your identity will remain anonymous but your review may still be made public.

**Do you want your identity to be public for this peer review?**  For information about this choice, including consent withdrawal, please see our Privacy Policy

Reviewer #1: No

Reviewer #3: No

**********

[NOTE: If reviewer comments were submitted as an attachment file, they will be attached to this email and accessible via the submission site. Please log into your account, locate the manuscript record, and check for the action link "View Attachments". If this link does not appear, there are no attachment files.]

To ensure your figures meet our technical requirements, please review our figure guidelines: https://journals.plos.org/plosone/s/figures

You may also use PLOS’s free figure tool, NAAS, to help you prepare publication quality figures: https://journals.plos.org/plosone/s/figures#loc-tools-for-figure-preparation.

NAAS will assess whether your figures meet our technical requirements by comparing each figure against our figure specifications.

# Author comment: Author response to Decision Letter 2

**DOI:** 10.1371/journal.pone.0360433.r004

8 Sep 2026

Response to Journal Requirement 1 (Reviewer-recommended citation):

We reviewed the suggested reference. However, as its focus on clinical nutritional adequacy in women with diagnosed binge eating disorder falls outside the scope of our study, we have elected not to include it, in line with the journal's guidance that there is no requirement to cite reviewer-recommended works unless directed by the editor.

Response to Journal Requirement 2 (Reference list check):

We have reviewed all cited references against the Retraction Watch database and PubMed retraction notices. No retracted articles were identified. All references remain valid and active in the literature.

Response to Reviewer 3, Comment 1 (Causal language in abstract):

We agree with this observation and have revised the final sentence of the abstract to use explicitly associative language in keeping with the cross-sectional nature of the study.

Response to Reviewer 3, Comment 2 (AUC undefined in abstract):

We thank the reviewer for catching this. AUC has been expanded to its full form on first use in the abstract.

Response to Reviewer 3, Comment 3 (Eating disorder exclusion as limitation):

We agree this warrants acknowledgment and have added a sentence to the Limitations paragraph addressing how this exclusion criterion may have attenuated the observed associations.

Response to Reviewer 3, Comment 4 (Pilot study results not reported):

To clarify, the pilot was conducted informally to assess item comprehension and questionnaire flow, and formal psychometric results were not recorded at that stage. Findings were used directly to guide wording revisions to selected items. The Methods section has been updated to reflect this accurately.

Response to Reviewer 3, Comment 5 (Causal language in Discussion):

We thank the reviewer for this careful reading and agree. We have reviewed the full Discussion and revised all instances of causal language to use consistently associative phrasing throughout.

Response to Reviewer 3, Comment 6 (Suggested reference):

Addressed in our response to Journal Requirement 1 above.

# Peer review: Decision Letter 2

**DOI:** 10.1371/journal.pone.0360433.r005

**Authors:** Junzheng Yang

27 Sep 2026

Association between Habitual MSG Consumption and Compulsive Eating Behaviors in University Students: A Cross-Sectional Study

PONE-D-26-22168R2

Dear Dr. Aljabry,

We’re pleased to inform you that your manuscript has been judged scientifically suitable for publication and will be formally accepted for publication once it meets all outstanding technical requirements.

Within one week, you’ll receive an e-mail detailing the required amendments. When these have been addressed, you’ll receive a formal acceptance letter and your manuscript will be scheduled for publication.

An invoice will be generated when your article is formally accepted. Please note, if your institution has a publishing partnership with PLOS and your article meets the relevant criteria, all or part of your publication costs will be covered. Please make sure your user information is up-to-date by logging into Editorial Manager at Editorial Manager® and clicking the ‘Update My Information' link at the top of the page. For questions related to billing, please contact billing support.

If your institution or institutions have a press office, please notify them about your upcoming paper to help maximize its impact. If they’ll be preparing press materials, please inform our press team as soon as possible -- no later than 48 hours after receiving the formal acceptance. Your manuscript will remain under strict press embargo until 2 pm Eastern Time on the date of publication. For more information, please contact onepress@plos.org.

Kind regards,

Junzheng Yang

Academic Editor

PLOS One

Additional Editor Comments (optional):

Reviewers' comments:

Reviewer's Responses to Questions

**Comments to the Author**

Reviewer #3: All comments have been addressed

**********

2. Is the manuscript technically sound, and do the data support the conclusions?

Reviewer #3: Yes

**********

3. Has the statistical analysis been performed appropriately and rigorously?

Reviewer #3: Yes

**********

4. Have the authors made all data underlying the findings in their manuscript fully available?

The PLOS Data policy

Reviewer #3: Yes

**********

5. Is the manuscript presented in an intelligible fashion and written in standard English?

Reviewer #3: Yes

**********

Reviewer #3: (No Response)

**********

what does this mean?). If published, this will include your full peer review and any attached files.

If you choose “no”, your identity will remain anonymous but your review may still be made public.

**Do you want your identity to be public for this peer review?**  For information about this choice, including consent withdrawal, please see our Privacy Policy

Reviewer #3: No

**********

# Editor report: Acceptance letter

**DOI:** 10.1371/journal.pone.0360433.r006

**Authors:** Junzheng Yang

PONE-D-26-22168R2

PLOS One

Dear Dr. Al Jabry,

I'm pleased to inform you that your manuscript has been deemed suitable for publication in PLOS One. Congratulations! Your manuscript is now being handed over to our production team.

At this stage, our production department will prepare your paper for publication. This includes ensuring the following:

* All references, tables, and figures are properly cited

* All relevant supporting information is included in the manuscript submission,

* There are no issues that prevent the paper from being properly typeset

You will receive further instructions from the production team, including instructions on how to review your proof when it is ready. Please keep in mind that we are working through a large volume of accepted articles, so please give us a few days to review your paper and let you know the next and final steps.

Lastly, if your institution or institutions have a press office, please let them know about your upcoming paper now to help maximize its impact. If they'll be preparing press materials, please inform our press team within the next 48 hours. Your manuscript will remain under strict press embargo until 2 pm Eastern Time on the date of publication. For more information, please contact onepress@plos.org.

You will receive an invoice from PLOS for your publication fee after your manuscript has reached the completed accept phase. If you receive an email requesting payment before acceptance or for any other service, this may be a phishing scheme. Learn how to identify phishing emails and protect your accounts at https://explore.plos.org/phishing.

If we can help with anything else, please email us at customercare@plos.org.

Thank you for submitting your work to PLOS One and supporting open access.

Kind regards,

PLOS One Editorial Office Staff

on behalf of

Director Junzheng Yang

Academic Editor

PLOS One
